# Poly(3-hydroxypropionate): Biosynthesis Pathways and Malonyl-CoA Biosensor Material Properties

**DOI:** 10.3389/fbioe.2021.646995

**Published:** 2021-03-04

**Authors:** Albert Gyapong Aduhene, Hongliang Cui, Hongyi Yang, Chengwei Liu, Guangchao Sui, Changli Liu

**Affiliations:** ^1^Key Laboratory of Saline-Alkali Vegetation Ecology Restoration, Northeast Forestry University, Ministry of Education, Harbin, China; ^2^College of Life Sciences, Northeast Forestry University, Harbin, China

**Keywords:** poly(3-hydroxypropionate), biosynthesis, cell factory, malonyl-CoA biosensor, life cycle assessment, material/physical property

## Abstract

Many single-use non-degradable plastics are a threat to life today, and several polyhydroxyalkanoates (PHAs) biopolymers have been developed in the bioplastic industry to place petrochemical-based plastics. One of such is the novel biomaterial poly(3-hydroxypropionate) [poly(3HP)] because of its biocompatibility, biodegradability, and high yield synthesis using engineered strains. To date, many bio-polymer-based functional composites have been developed to increase the value of raw microbial-biopolymers obtained from cheap sources. This review article broadly covers poly(3HP), a comprehensive summary of critical biosynthetic production pathways comparing the yields and titers achieved in different Microbial cell Factories. This article also provides extensive knowledge and highlights recent progress on biosensors’ use to optimize poly(3HP) production, some bacteria host adopted for production, chemical and physical properties, life cycle assessment for poly(3HP) production using corn oil as carbon source, and some essential medical applications of poly(3HP).

## Key Points

1. Poly(3HP) is a promising alternative to petro-diesel based plastic.
2. β-alanine biosynthetic pathway is efficient in producing poly(3HP).
3. Malonyl-CoA biosensor increases poly(3HP) yield.

## Introduction

Poly(3-hydroxypropionate) [poly(3HP)] is a promising monomer of the polyhydroxyalkanoate (PHA) family of material properties similar to other short chain lengths (SCL) PHAs like poly(3-hydroxybutyrate) [poly(3HB)]. It is a well-characterized polyester, biodegradable, biocompatible, and has excellent mechanical properties such as high flexibility, high tensile strength, and high rigidity but more stable than polylactic acid (Andreeßen et al., 2010, 2014b). Poly(3HP) homopolymer biosynthesis came to the limelight in 2010, using glycerol as a carbon source in a two-step fed-batch fermentation and proved to be a promising alternative petrochemical-derived plastic.

Within half a century, glycerol, a by-product of biodiesel production, has become a common carbon source for several biotechnological research in the world. Due to environmental pollution associated with the petrochemical industry resulting in climate change and overexploited fossil reserves, there needs to use biological approaches through metabolic engineering to produce many chemicals from non-renewable sources using inexpensive and abundant raw materials (Keasling, 2010).

The effectiveness and success of the biological production of a platform chemical like 3-hydroxypropoic acid (3-HP) are dependent on the development of microbial Cell Factories; thus prompting vigorous and extensive researches conducted in diverse fields such as biosynthesis, gene amplification, engineering of enzymes, design of the genetic circuit, genome editing, bioinformatics, adaptive laboratory evolution, multiomics using suitable hosts, and biosynthetic pathways of microorganisms through the process called “Design-Build-Test-Learn (DBTL)” cycles (Li et al., 2012; Abatemarco et al., 2013; Cheong et al., 2015; Wu et al., 2016). Furthermore, to commercialize these processes through effective biosynthetic pathways to achieve economically viable products, biosensors must be constructed in a suitable metabolic route, optimized to produce desirable results (Liu et al., 2015a).

In order to increase yields of PHAs through synthetic biotechnology and metabolic engineering, there has been the use of metabolite biosensors to detect and monitor a target metabolite *in vivo* by high-throughput screening (HTS) (Eggeling et al., 2015). Although previous review papers have discussed some biosynthetic pathways, material and chemical characteristics of poly(3HP) (Andreeßen et al., 2014b; Chang et al., 2018), however, this review extensively discusses up to date the metabolic pathways that can be used to produce poly(3HP) from glycerol and glucose. The paper also focuses on the production host, the use of malonyl-CoA biosensors to optimize yield, life cycle assessment for poly(3HP) production using corn oil as a carbon source, and the physical and chemical properties. Finally, we will highlight some applications of poly(3HP).

### PHAs

Polyhydroxyalkanoates are classes of intracellular, linear high molecular aliphatic bacterial storage polyesters, which are biocompatible thermoplastics and biodegradable into carbon dioxide and water by many microorganisms (Zhang et al., 2018). Physical or material properties of PHAs are similar to petroleum-derived plastics but exhibit some different characteristics ranging from brittleness to flexibility and elasticity, as shown in Table 1 (Muhammadi et al., 2015). PHA polymers exhibition of features such as elasticity, flexibility, biodegradability, and renewability are highly dependent on these factors, namely; (1) their biosynthesis pathways of production (Masood et al., 2014), (2) monomeric composition (Luzi et al., 2019), and (3) chemical structure (Bugnicourt et al., 2014).

**TABLE 1:** Comparison of thermodynamic properties of poly (3-HP) compared to other PHAs copolymers.

PHAs Type	Thermal performance			Mechanical behavior			References
		
	*T*_m_ (^*o*^C)	Δ*H*_m_ (J/g)	*T*_g_ (^*o*^C)	*E* (MPa)	σ*_mt_* (MPa)	ε*_b_* (%)	
Poly(3HP)	79.1	64	−20	0.3	27	634	Zhou et al., 2011
Poly(3HB)	177	88	3	3.5	37	6	Chen and Wu, 2005
Poly(3HV)	119	−16	−15		31	14	Zhao et al., 2014
Poly(4HB)	53	−	−45	149	104	1000	Zhou et al., 2011
Poly-lactic acid	164	57	62	2.0	55	5	Zhao et al., 2014; Zhou et al., 2011
PP	174	148	−13	1.6	34	400	Zhao et al., 2014; Zhou et al., 2011
PS	110		100	3.7	54	4	Chen and Wu, 2005

Furthermore, the production of PHAs is currently not economical in comparison to that of synthetic plastics. To make bacterial PHAs production cost-effective, a few critical factors need to be addressed, such as screening and selection of potential bacterial strains (Kumar et al., 2016, 2020), synthesis pathways, advanced tools and technologies (Możejko-Ciesielska and Mostek, 2019), carbon and nitrogen source (Zahari et al., 2014), and cost-efficient down-stream pro-cesses (Koller and Braunegg, 2018). The purposeful application of the PHAs is also a crucial factor determining its importance and economy (Chen et al., 2017; Cao et al., 2019).

Many researchers have channeled their interest in the production of PHAs recently due to inexpensive carbon sources as substrate and the dangerous problem associated with single-use plastic waste disposal (Chen and Patel, 2012).

Previously, ring-opening polymerization (ROP) of β-propiolactone and 3-HP ester condensation was the chemical processes for synthesizing poly(3HP). There has been no evidence of a natural organism capable of synthesizing it (Wang et al., 2012, 2013; Heinrich et al., 2013).

However, poly(3HP) was not commercially feasible to produce in large quantities because β-propiolactone as a substrate has carcinogenic properties (Dunn, 2012; Dunn et al., 2016). 3-HP, which has low crystallinity, is an ideal precursor that forms a constituent for several copolymers like poly(3-hydroxybutyrate-co-3-hydroxypropionate) [poly(3HB-co-3HP)] or poly(3-hydroxypropionate-co-4-hydroxybutyrate) [Poly(3HP-co-4HB)] (Zhou et al., 2011; Wang et al., 2013).

3-hydroxypropoic acid, acrylate, and 1,3-propanediol (1,3-PDO) were previously utilized as dependent precursors for the biosynthesis of poly(3HP) and 3-HP-containing copolymer (Gao et al., 2014). However, these expensive precursors and Vitamin B_12_ increased poly(3HP) production cost making it economically not feasible.

Artificial pathways were constructed for poly(3HP) biosynthesis from inexpensive carbon sources such as glucose and glycerol without the addition of precursors to remedy the challenges arising from high production costs (Andreeßen et al., 2010). Nevertheless, only 13 mg/L poly(3HP) was produced by recombinant *Escherichia coli* strain using glucose as a substrate. Andreeßen et al. (2010) produced 1.42 mg/L poly(3HP) by glycerol conversion using a recombinant *E. coli* strain carrying glycerol dehydratase of *Clostridium buytricum DhaB1Cb*, propionaldehyde dehydrogenase of *Salmonella enterica serovar Typhimurium* LT2 (*PduPSe*), and PHA synthase gene of *Ralstonia eutropha* HI6 (*PhaC1Re*) in a two-step fed-batch fermentation process (Andreeßen et al., 2010).

## Metabolic Pathways for the Synthesis of Poly(3HP)

Several microorganisms have been reported to artificially produce poly(3HP) through microbial genetic modification using various routes and different substrates such as glucose and glycerol with the addition of some precursors (Wang et al., 2014).

### Traditional Pathways

In the traditional routes of biosynthesis of poly(3HP), there is dehydration of glycerol in the propionaldehyde dehydrogenase route (PduP route) 3-HP route. However, there is no such dehydration of glycerol in the Malonyl-CoA route, and there is no restriction concerning the use of a specific carbon source.

### The PduP Route

In 2010, the first known route for biosynthesis of poly(3HP) was established and composed of these three steps: (i) dehydration of glycerol (ii) 3-hydroxypropionaldehyde (3-HPA) oxidation, and CoA ligation to 3-hydroxypropionyl-CoA (3-HP-CoA), and (iii) polymerization of 3-HP-CoA to poly(3HP) (Andreeßen et al., 2010). In recent years, glycerol, a by-product of biodiesel production, has become the most attractive carbon source for synthesizing various biological products. Its availability, however, exceeded the demand for regular consumption in the chemical, pharmaceutical, and cosmetic industries. Interestingly, in the transesterification of vegetable oil or animal fat with methanol, 10% of the product is glycerol (Quispe et al., 2013), causing a sharp drop in glycerol price. Therefore, poly(3HP) synthesis from cheap glycerol can reduce costs and improve economic efficiency.

A two-step fed-batch fermentation process was used in 2010 for the synthesis of poly(3HP); the cells were maintained and propagated in an anaerobic phase with a reducing agent, which then accumulates 1.4 g/L of poly(3HP), accounting for 12% of the cell dry weight (Table 2; Andreeßen et al., 2010). Upon further analysis, Andreeßen et al. (2010) concluded that the *DhaB1Cb* is only active under strictly anaerobic conditions. The maintenance of anaerobic conditions is achieved by reducing agents (disodium fumarate and sodium potassium tartrate), increasing the production cost, and producing a relatively low yield (Andreeßen et al., 2010).

**TABLE 2:** Biosynthesis of poly (3-HP) by recombinant microorganisms in different pathways.

Host	Pathway	Poly (3-HP)% in cells	Cell density (g/L)	Cultivation time (h)	Substrate	Cofactor/inducer/antibiotic(s)	References
*E. coli*	β-alanine	39.1	10.2	72	Glucose/Glycerol	None/IPTG/Amp^*E*^,Cm^*R*^	Lacmata et al., 2017
*E. coli*	β-alanine	10.2	0.5	72	Glucose/Glycerol	None/IPTG/Amp	Wang et al., 2014
*E. coli*	Malonyl-CoA	0.98	1.32	72	Glucose	Biotin/IPTG/Amp,Cm	Wang et al., 2012
*E. coli*	PduP	12	1.44	92	Glycerol	None/IPTG/Km	Andreeßen et al., 2010
*E. coli*	PduP	46.4	21.8	84	Glycerol	VB_12_/IPTG/Amp,Cm	Wang et al., 2013
*E. coli*	PduP	67.9	36	48	Glycerol	VB_12_/IPTG/none	Gao et al., 2014
*K. pneumoniae*	3-HP	12.7	0.24	48	Glycerol	None/L-arabinose/Km,Cm	Feng et al., 2015
*E. cole*	3-HP	92	5.5	48	1,3PD	None/none/Amp,Km	Zhou et al., 2011
*E. coli*	3-HP	57	20	28	1,3PD	None/none/Amp,Km	Zhou et al., 2011
*E. coli*	3-HP	9.8	3	72	Glycerol	None/IPTG/Km	Heinrich et al., 2013

In another study, a bacterial strain of high yielding poly(3HP) ability was developed using a fermentation process in the presence of vitamin B_12_-dependent glycerol dehydratase gene *dhaB123Kp* and the helper gene (*gdrABKp)* in *Klebsiella pneumonia* with low oxygen sensitivity to replace *DhaB1* from *Salmonella typhimurium*. The propionaldehyde dehydrogenase gene (*pduPSt)* of *S. typhimurium* and the *phaC1Re* was expressed in *E. coli* (Wang et al., 2013).

**FIGURE 1:**
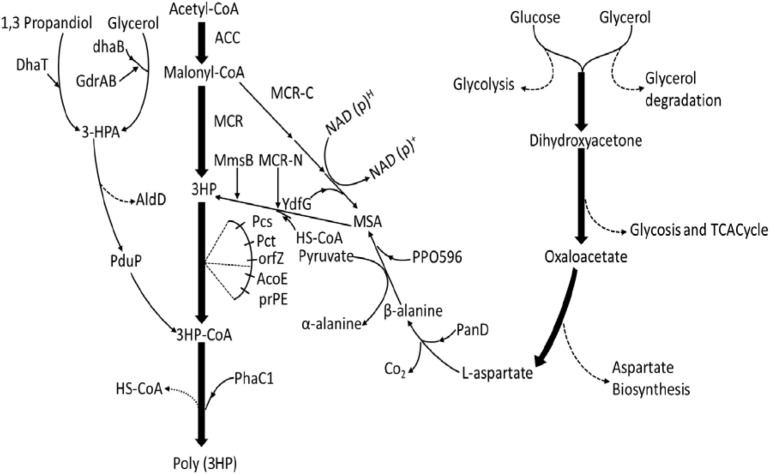
Overview of the four biosynthetic poly(3HP) production pathways from different engineered strains with different starting substrates. From 1, PduP route (– – – (DhaB and GdrAB, glycerol dehydratase; PduP, PhaC1.3-HP route (—–): poly(3HP) synthesis from glycerol and 1, 3-propanediol. DhaB and GdrAB, glycerol dehydratase; Dhat, AldD, Pcs’ (ACS), Pct, PhaC1. Malonyl-CoA route (– – –): poly(3HP) synthesis from Acetyl-CoA, Acc, MCR, PrpE, Pcs’, PhaC1. β-alanine route (– – –): poly(3HP) synthesis from glucose and glycerol. Aspartate biosynthesis, PanD, PP0596, YdfG, PrpE, PhaC1 (Steinbüchel and Lütke-Eversloh, 2003; Andreeßen et al., 2010, 2014b; Wang et al., 2012, 2013; Feng et al., 2015; Lacmata et al., 2017).

In the optimization strategy, the poly(3HP) anaerobic accumulation phase in the experiment was omitted by not adding disodium fumarate and sodium-potassium tartrate reducing agents. This method, therefore, reduced the production cost while lowering the fermentation process and additives. Also, the addition of vitamin B_12_ maintained the activity of glycerol dehydratase in the metabolic pathway. Glucose is also added as an adjuvant to convert the biomass energy and glycerol produced by glucose catabolism into poly(3HP) production, thereby increasing yield. The synthesis method only uses aerobic fermentation to produce a poly(3HP) yield of 21.8 g/L, accounting for 46.4% of the cell dry weight (Table 2 and Figure 1; Wang et al., 2013). Although the glycerol synthesis of poly(3HP) has made significant progress, there are still some unresolved challenges. For instance, the addition of vitamin B_12_ leads to an increase in the cost of fermentation, which can be reduced by using *K. pneumonia* strain, which produces vitamin B_12_ as the host strain for poly(3HP) synthesis.

Again, the redox balance problem in fermentation production also plaques the poly(3HP) synthesis, which may be omitted by introducing an anabolic pathway that overcomes NADH (Andreeßen et al., 2014a). Another alternative to vitamin B_12_ was *Shimwellia blattae*, a natural producer of vitamin B_12,_ as a vector. The vector was engineered to express a 1,3-propanediol (PDO) dehydrogenase gene (*dhaTPp)* and aldehyde dehydrogenase (*aldDPp*) of *Pseudomonas putida* (Heinrich et al., 2013). The *aldDPp*, *Clostridium propionicum* X2 propionate coenzyme A transferase gene (*pctCp)*, and *R. eutropha* H16 *phaC1Re* (Figure 1) can produce a high yield of poly(3HP) in the absence of vitamin B_12_. However, contrary to the two-phase fermentation method described in some research studies, the fermentation process uses anaerobic-aerobic two-stage culture, which not only achieves higher cell dry weight than complete anaerobic production but also reduces the conversion of the precursor 3-hydroxypropionaldehyde to 1,3-PDO during the aerobic phase (Andreeßen et al., 2010). Our previous studies investigated the influence of different oxygen supply patterns on poly(PHB) yield and bacterial community diversity using domestic sewage sludge and produced a poly(PHB) yield of 64% in cell dry weight of sequential batch reactor (SBR1) and 53% in SBR2. Again, the poly(PHB) synthesis from activated sludge and the effect of chemical oxygen demand (COD)/N ratio on poly(PHB) accumulating ability in an anaerobic/aerobic cycle SBR was also investigated. Poly(PHB) produced reached a maximum of 64.2% of cell dry weight when COD was 1,200 mg/L, and COD/N/P was 1,200/9.6/30, respectively (Liu et al., 2013, 2016b).

Another exciting outcome is the increase in cell dry weight during fermentation with crude glycerol, which can also be associated with the organic matter in the crude glycerol carbon source can be utilized as a supplemental carbon source by microorganisms, resulting in higher biomass than pure glycerol fermentation (Andreeßen et al., 2014b). The final results showed that in the 2 L fed-batch fermentation reactor, poly(3HP) accumulation accounted for 9.8 ± 0.4% of the cell dry weight after 72 h of culture (Table 2; Heinrich et al., 2013). The introduction of the propionyl-CoA synthetase gene *prpEEc* of *E. coli* into the *K. pneumonia* strain (Figure 1) resulted in aeration conditions that play a crucial role in poly(3HP) production and cell growth. Among the engineered metabolic pathways studied by Feng et al. (2015), 3-HP was used as a poly(3HP) precursor. However, poly(3HP) production accounted for only 10% of 3-HP production under three aeration conditions with different shaking culture rates of 50, 100, and 200 r/min (Feng et al., 2015).

Finally, under the condition of no vitamin B_12_ and optimized aeration, poly(3HP) content accounted for 12.7% of the cell dry weight after 48 h of culture. It is speculated that the low conversion efficiency of poly(3HP) from 3-HP is probably due to the low enzyme activity of propionyl-CoA synthetase (*PrpE*), and a similar phenomenon was also found when constructing the *R. eutropha* expression vector (Fukui et al., 2009).

On the other hand, *PrpE* is a crucial enzyme for propionyl-CoA catalytic synthesis in propionate catabolism. Compared to propionate, 3-HP may not be suitable as a *PrpE* synthetic substrate. Improvement of poly(3HP) production can be achieved by modifying *PrpE* or using other *PrpE* sources such as *S. typhimurium* (Lee et al., 2005). Generally, there is a balance between molecular weight and PHA polymers yield (Lim et al., 2011). The molecular weight and yield of PHA polymers are generally determined by the ratio of the three key enzymes that synthesize PHA, namely the *phaC*-encoded PHA synthase, the *phaA*-encoded β-ketothiolase, and the *phaB*-encoded NADPH-dependent acetoacetyl-CoA reductase. Thus, the expression levels of these three genes may lead to differences in PHA synthesis yield. It is well known that for genes downstream of the operon, genes close to the promoter will be highly expressed (Sim et al., 2001).

Furthermore, researchers explored the effect of the *phaCAc* operon gene sequence on the targeted gene’s transcription efficiency in the engineered *E. coli* (Hiroe et al., 2012). It was evidenced that the specific location and the sequencing of the nucleic acid sequence in the operon constitute an effective method for regulating gene expression. For example, changing the promoter or adjusting the inducer concentration can effectively control the yield and the molecular weight of the synthetic PHA polymer synthesized by the recombinant strain. On this account, Andreeßen et al., 2014a) rearranged the *phaCAc* operon gene sequence to optimize the poly(3HP) synthesis operon structure by engineering a carbon-dependent plasmid in a triosephosphate isomerase knockout mutant.

The plasmid addition system overcomes the problem of plasmid loss during fermentation, thereby increasing the yield of the polymer from 52 ± 3.3 to 57 ± 1.6% (wtPHA/wtCDW) in recombinant *E. coli* (Table 2; Zhou et al., 2011). The metabolic pathway synthesis of poly(3HP) yields nearly a two-fold increase while at the same time obtaining high value-added by-products. To stabilize poly(3HP) production, Gao et al. (2014) constructed a genetically stable recombinant strain of *E. coli*. Based on amino acid synthesis and metabolism, chromosomal gene integration and plasmid addiction system technology were used to obtain high-yield poly(3HP) engineering strain *E. coli* Q1738 (Gao et al., 2014).

Under aerobic conditions, the recombinant strain yielded 25.7g/L poly(3HP) by glycerol fermentation, and the cell accumulation was as high as 67.9% (Table 2; Andreeßen et al., 2014b; Gao et al., 2014). This study shows that if two or more types of plasmids are present in the same strain, plasmid stability will be reduced, resulting in a low yield of poly(3HP). It has been reported that in the stability test of the verified plasmid, the stability decrease as the length of the plasmid increases, and the metabolic load caused by the rise in the number of plasmid repeats is the leading cause of plasmid loss. The multiplex plasmid has a substantial metabolic burden on the cells, and the single plasmid is more reasonable and stable. Furthermore, the toxic effects of 3-hydroxypropionaldehyde produced by glycerol dehydratase conversion in the glycerol pathway are also reasons for reducing poly(3HP) production.

### Malonyl-CoA Pathway

The Malonyl-CoA route is the *de novo* synthesis of fatty acids. This pathway has no restrictions on the use of various carbon sources. The substrate can be metabolized by cells to produce acetyl-CoA, thereby further creating the intermediate product Malonyl-CoA. The goal of synthesizing the final product poly(3HP) can be achieved, which is the preferred route for the low-cost synthesis of poly(3HP) (Wang et al., 2013).

The pathway shows how acetyl-CoA is converted into Malonyl-CoA by the acetyl-CoA carboxylase gene (*acc*). A Malonyl-CoA reductase gene (*mcrCa)* of *Chloroflexus aurantiacus* was then introduced to synthesize 3-HP. The propionyl-CoA synthetase gene (*pcsCa)* was then transferred into *R. eutropha* to synthesize poly(3HP) (Fukui et al., 2009). This study successfully established a new artificial pathway using a cheap carbon source to synthesize poly(3HP) (Figure 1). In the Malonyl-CoA route, the *acc* genes were overexpressed to increase the intracellular Malonyl-CoA concentration and simultaneously transferred propionyl-CoA into *E. col* to increase Poly(3HP) accumulation. The synthetase gene (*prpEEc)* replaces the *pcsCa* gene and reconstitutes the poly(3HP) synthesis pathway in *E. coli*. The recombinant *E. coli* synthesizes poly(3HP) with glucose as the carbon source yielding 13 mg/L, accounting for 0.98% of the cell dry weight (Table 2; Wang et al., 2012; Andreeßen et al., 2014b).

In the Malonyl-CoA pathway, when the poly(3HP) synthesis was carried out in *E. coli* with a transparent genetic background and cultured, the non-structural-related carbon source could be utilized, but its yield was significantly too low and might be caused by plasmid loss (Wang et al., 2014).

Firstly, the plasmid-selective marker gene containing antibiotic resistance in the recombinant strain is secreted in the medium, which may cause rapid decomposition of the corresponding antibiotic and excessive growth of plasmid-free cells. Secondly, the malonyl-CoA reductase (MCR) of *C. aurantiacus* catalyzes the conversion of malonyl-CoA to 3-HP. It is a critical enzyme in poly(3HP) microbial production, while MCR’s toxic effect of the intermediate malonate semialdehyde and the host cell *E. coli*. Thirdly, MCR’s low activity is also one reason for the low poly(3HP) content. MCR enzyme from phototrophic *C.aurantiacus* is the focal enzyme in microbial production with an optimal enzyme reaction temperature of 57°C (Hügler et al., 2002), which is higher than the optimal growth temperature of the carrier *E. coli* and the culture medium, which is 37°C. Under this condition, MCR enzyme activity can only reach 60% of the full process, so the culture temperature becomes a factor that restricts MCR’s operation. Moreover, MCR enzymatic process in *E. coli* is tightly controlled by its physiological and environmental characteristics, which may be affected when any of these factors change (Hügler et al., 2002).

A detailed analysis of the MCR functional domain was investigated to solve the low catalytic activity of MCR in the Malonyl-CoA pathway. The MCR enzymatic activity has two active centers. Namely, Malonyl-CoA reductase N-terminal (MCR-N) (amino acids 1–549) and Malonyl-CoA reductase C-terminal (MCR-C) (amino acids 550–1,219). These two centers catalyze the synthesis of malonyl CoA 3-HP in two steps; MCR-C catalyzes the malonyl CoA reduction to the free intermediate malonic acid semialdehyde, catalyzed 3-HP by MCR-N (Figure 1). The activity of key enzymes in the Malonyl-CoA pathway was analyzed by Liu et al. (2016a), and it was found that the MCR-C process was 4–5 times lower than MCR-N; that is, the imbalance of MCR enzyme activity restricted the efficient accumulation of downstream products. Targeted mutagenesis of MCR-C and MCR-N was carried out to regulate their expression rate, and the fermentation conditions were optimized. The yield of poly(3HP) precursor 3-HP was up to 40.6 g/L (Liu et al., 2016a).

Several studies have assessed the accumulation of polyketides in the 3-HP pathway by dynamic regulation Malonyl-CoA system. When the intracellular supply of Malonyl-CoA was sufficient, downstream products’ synthesis capacity increased by 100 times (Yuzawa et al., 2012). Furthermore, Zhang et al. (2012) proposed designing a dynamic regulation system for Malonyl-CoA to improve the synthesis and conversion rate of Malonyl-CoA, thereby increasing the yield of the final product (Zhang et al., 2012).

### Modified Pathway

The modified or the current route for the biosynthesis of poly(3HP) was developed, employing β-alanine as intermediate, thereby named the β-alanine pathway.

### B-Alanine Pathway

Poly (3-HP) accumulation can be higher in the *PduP* route, but maintaining glycerol dehydratase activity requires an exogenous supply of vitamin B_12_, resulting in increased production costs. Furthermore, in the Malonyl-CoA pathway, when glucose was used as a sole carbon source, only 13 mg/L poly(3HP) was accumulated by the *E. coli* (Wang et al., 2012). The biosynthesis of poly(3HP) from glucose as the only carbon source has been reported to give a lower yield, and to produce commercially on a large scale is not feasible. Hence, a new way to synthesize poly(3HP) from β-alanine as an intermediate in the recombinant *E. coli* to mitigate these problems (Figure 1; Wang et al., 2014). Lacmata et al., 2017, after a series of system optimization, was able to cloned and amplified these genes simultaneously; *panDEc* from *E*. *coli*, with its mature factor *panMEc*, 3-hydroxy acid dehydrogenase gene (*ydfG)*, and propionyl-CoA synthetase gene (*prpEEc*) (Lacmata et al., 2017). *P. putida*’s β-alanine-pyruvate transaminase gene (*pp0596)* and *phaC1Re* were expressed in *E. coli.* Using glycerol and glucose as carbon sources, the poly(3HP) content in the recombinant strain was 0.5 g/L, which was 10.2% of the cell dry weight (Table 2; Wang et al., 2014). Although the poly(3HP) content was low, the advantage of this pathway over other reported routes is redox neutrality, the non-addition of coenzymes and expensive precursors, and the use of a wide range of carbon sources. The above advantages are also worthy of further research and development by researchers.

A new strategy utilized β-alanine as an intermediate was constructed (Wang et al., 2014). It was observed that the L-aspartate produced from the aspartic acid biosynthesis pathway was converted into β-alanine utilizing L-aspartate -α-decarboxylase (PanD) of *E. coli. The*β-alanine was further converted into malonate semialdehyde by β-alanine-pyruvate transaminase of *P.putida*. Furthermore, the malonate semialdehyde was reduced by YdfG from *E. coli* to 3-HP as a precursor to poly(3HP) (Lacmata et al., 2017; Figure 1). The experimental process found that insufficient supply of β-alanine restricted the biosynthesis of poly(3HP), possibly due to low L-aspartate-α-decarboxylase activity or low intracellular L-aspartate concentration L-aspartate-α-decarboxylase is the rate-limiting enzyme in the β-alanine pathway and is transferred to PanD of *E. coli* or glutamate rod *Corynebacterium glutamicum*. The enzyme increases the synthesis efficiency to produce a sufficient amount of beta-alanine. However, the experimental results showed that the increase in L-aspartic acid concentration is not adequate for the conversion of β-alanine. The final experiment showed that recombinant *E. coli* under suitable conditions synthesized 10.2 g/L and 39.1% (wt/wt [cell dry weight] of poly(3HP), respectively, without the addition of vitamin B_12_ in flask culture and fed-batch fermentation, respectively (Table 2; Lacmata et al., 2017).

## The Production Host of Poly(3HP)

Some microorganisms are to produce poly(3HP) by engineering microbes, such as bacteria and fungi, as shown in Table 3 (Jers et al., 2019). Choosing the appropriate host to use involves several factors. The microbes must have the ability to tolerate organic acids as well as potentially toxic impurities in carbon sources. As one of the broadly used substrates, glycerol dehydratase is coenzyme B_12_-dependent; the production host should preferentially synthesize coenzyme B_12._

**TABLE 3:** Overview of some bacterial species used for the production of poly(3HP).

Bacterial species	Natural producer	B_12_ synthesis	References
*Klebsiella pneumonia*	Yes	Yes	Feng et al., 2015; Wang et al., 2013
*Escherichia coli*	No	No	Gao et al., 2014
*Bacillus Subtilis*	No	No	
*Shimwellia blattae*	No^*a*^	Yes	Heinrich et al., 2013
*Lactobacillus reuteri*	Yes	Yes	Linares-Pastén et al., 2015
*Ralstonia eutropha*	Yes	No	Fukui et al., 2009

*^*a*^Naturally produces 3-HPA.*

### Genetic Modification of Bacteria in Poly(3HP) Production

#### Escherichia coli

*Escherichia coli* is the predominately used bacteria for metabolic engineering to produce a wide range of valuable compounds like biofuels, chemicals, polymers, and proteins, and the production procedures often depend on the expression of different genes carried by plasmid vectors (Kroll et al., 2010). It has been broadly used as chassis for the production of poly(3HP). In 2010, a recombinant *E. coli* strain was used as a host bacteria to produced 1.42 g/L poly(3HP) in the fed-batch fermentation. The process employed crude glycerol as the substrate by introducing the glycerol dehydratase of *C.butyricum*, PduP of *S.entarica* serovar *Typhimurium* LT2, and PhaC1 of *R.eutropha* into the *E. coli* (Andreeßen et al., 2010). A new strategy was developed that accumulated poly(3HP) using glycerol as a substrate by employing glycerol dehydratase of *K. pneumonia*, which is functional along with its reactivate *GdrABL* under both aerobic and anaerobic conditions and has been used in the production of 1,3-PDO and 3-HP (Huang et al., 2012). Furthermore, a recombinant strain of *E. coli* under optimal conditions accumulated the highest CDW of 21.8 g/L containing 46.4% of poly(3HP) without any expensive precursor during aerobic fed-batch fermentation (Wang et al., 2013).

#### Klebsiella pneumonia

It is a pathogenic bacteria sparingly used in poly (3-HP) production compared to engineered *E. coli* strains because of the low accumulation of poly (3-HP). Engineered *K. pneumonia* can naturally produce vitamin B12, making it economically advantageous compared to *E. coli*, which lacks the coenzyme vitamin B_12_ (Ashok et al., 2013). Under controlled aeration, engineered strain Q61643 accumulated 0.24 g/L of poly(3HP), which accounted for 12.7% of the cell dry weight (Feng et al., 2015). Again, using the vitamin B_12_ dependent glycerol dehydratase from *K. pneumonia*, which is less sensitive to oxygen, a higher poly(3HP) was achieved, accumulating 46.4% of the cell dry weight (Wang et al., 2013).

The strategy employed by Wang et al. (2013) was improved by combining chromosomal gene integration (genes involved in glycerol dehydration) and plasmid addiction system (synthase and CoA acylating enzyme genes) based on tyrosine anabolism, and the resulting *K.pneumonia* strain accumulated 67.9% of poly(3HP) the cell dry weight (Gao et al., 2014).

Furthermore, a metabolically engineered strain of *K. pnuemonia* is used to produce 0.24 g/L Poly(3HP) from glucose, which accounted for 12.7% of cell dry weight without the external addition of the coenzyme vitamin B_12_ under controlled dissolved oxygen (100rmp, 100mL working volume in 250 mL non-baffled flasks) (Feng et al., 2015).

#### Shimwellia blattae

A member of the Enterobacteriaceae *S. blattae*, cobalamin forming, non-pathogenic, previously isolated from the cockroach *Blattae orientalis* genes *DhaT* and *AldT* of *P.putida* KT2442, *pct* of *Clostridium propionicum* X2, and *PhaC1Re* enabled the production of 0.26 g/L poly(3HP). The production of crude glycerol from biodiesel as the substrate without the addition of any co-factors such as vitamin B_12_ because it produces 1,3-PDO, a native coenzyme B_12_ –dependent glycerol dehydratase (Heinrich et al., 2013).

## Malonyl-CoA Biosensor Application in Improving Poly(3HP) Yield

**FIGURE 2:**
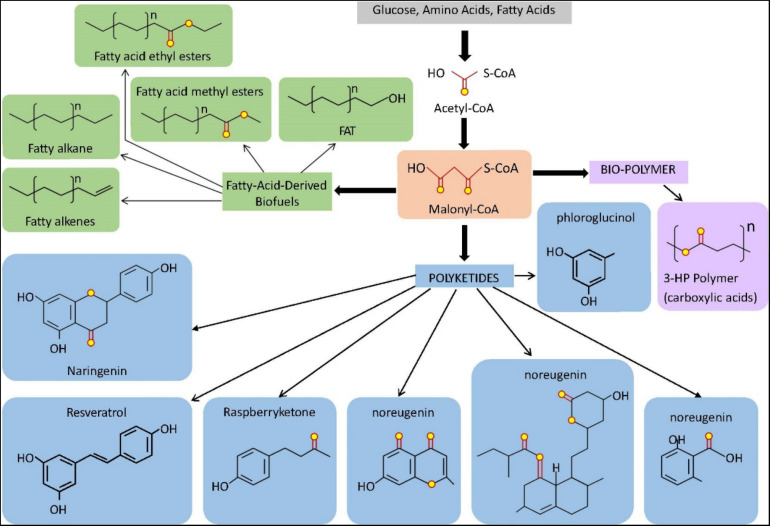
Overview of some compounds derived from the microbial synthesis of Malonyl-CoA dependent molecules. Malonyl-CoA is a direct product of acetyl-CoA that can be used as a common precursor for the biosynthesis of fatty acids-derived biofuels, biopolymers, and polyketides. The light orange box indicates Malonyl-CoA. Light green boxes indicate fatty acid-derived biofuel products and light blue boxes indicate polyketide products. The light purple box indicates bio-polymer (carboxylic acids). For fatty acids, n represent the number of Malonyl-CoA-derived C2 unit in the terminal methyl group and terminal carboxyl group which accounts for +1.

The biosynthesis industry has applied synthetic biology and metabolic engineering within the last decade by assembling biotechnological components to optimize production titer, rate, and microbial yield (Nielsen and Keasling, 2016; Yu et al., 2019). In industrial biotechnology, enormous interest in exploring intracellular Malonyl-CoA flux for the biosynthesis of fatty acids and non-fatty acid-based end chemicals in microbial cell factories, such as *E. coli* and *Saccharomyces cerevisiae* through the role of Malonyl-CoA as a precursor metabolite as shown in Figure 2 (Johnson, 2019). A reliable metabolic system with a fine-tuned gene expression can assist in achieving such optimization. Such a built system aims to reduce the metabolic waste burden and flux imbalance, and toxic accumulation on the cell (Jones et al., 2015). Alternatively, constructing a dynamic biosynthetic pathway employing metabolite biosensors can increase yield (Tan and Prather, 2017). A metabolite biosensor has been used in improving the production of 3-HP (David et al., 2016) and fatty acid ethyl esterification (Zhang et al., 2012) to maximize yields. Another study proved that over-expression of the *acc* gene led to a significant increase of poly(3HP) in a recombinant Poly(3HP) producing *E. coli* due to the increased intracellular pool of malonyl-CoA (Wang et al., 2012). Together, the studies confirmed that the overexpression of *acc* genes could enhance the cellular malonyl-CoA concentration.

The low intracellular concentration of Malonyl-CoA limits the efficient synthesis of downstream products. Eliminating the bottleneck constraints of Malonyl-CoA is the prerequisite basis for increasing the output of poly(3HP). It provides a theoretical basis for industrializing different products in low-cost and large-scale pharmaceutical and chemical fields. Previous research showed that low Malonyl-CoA production is the key factor restricting the efficient accumulation of poly(3HP). A recombinant bacterial capable of accumulating high amounts of poly(3HP) was developed.

The high or low concentration of Malonyl-CoA inhibits bacterial growth, thereby restricting poly (3HP) synthesis. Dynamic regulation of the synthesis and conversion rate of Malonyl-CoA to solve bacterial cytotoxicity was considered. Using wild-type bacteria can also regulate metabolism in real-time according to changes in the intracellular, extracellular environment. However, due to the lack of the necessary sensory control system, the engineered bacteria constructed with foreign genes cannot accurately control the metabolism according to the host’s state and synthesize corresponding enzymes sparingly, resulting in metabolic imbalance and resource waste. Therefore, the introduction of sensory regulation systems in engineering bacteria and the adjustment of metabolism according to the host state have an essential role in improving product synthesis (Ding et al., 2015; Mo et al., 2017).

**FIGURE 3:**
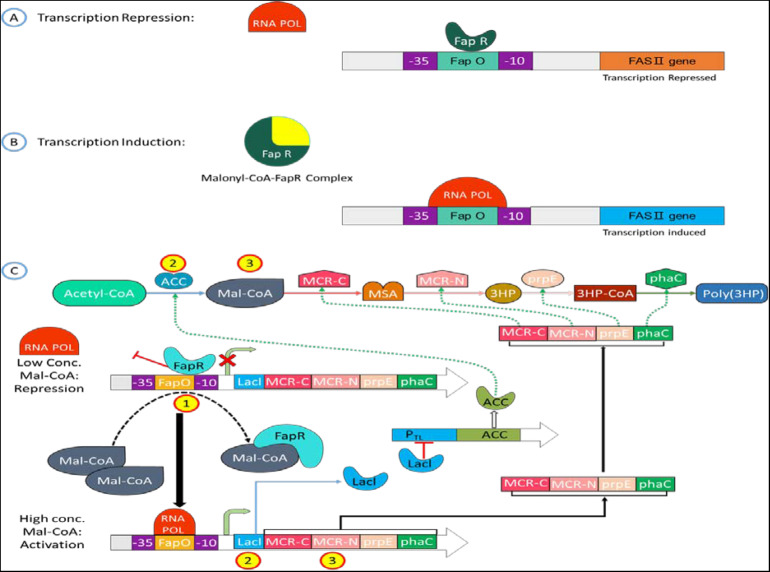
Mechanism of Malonyl-CoA sensing in a cell circuit. **(A, B)** Schematic representation of Malonyl-CoA metabolic sensor that shows binding of Malonyl-CoA to C-terminus of FapR cascade into a conformation change of N-terminus, FapR-fapO interaction is destabilized. **(C)** The metabolic switch turns on the expression of the Malonyl-CoA source pathway (ACC) and turns off the sink pathway (FAS) at a low cellular Malonyl-CoA amount. The switch turns on the sink pathway at a high cellular Malonyl-CoA amount and turns off the source pathway.

Recently, the FapR protein domain has been predicted (Albanesi et al., 2013; Albanesi and De Mendoza, 2016), and the mechanism by which FapR regulates fatty acid synthesis has also been elucidated (Ellis and Wolfgang, 2012; Figure 3). When the intracellular Malonyl-CoA concentration is low, FapR binds to FapO on the promoter causing Steric hindrance. It prevents RNA polymerase (RNA Pol.) from attaching to the promoter, thereby inhibiting downstream gene transcription; when Malonyl-CoA concentration is high, FapR forms a complex with Malonyl-CoA and detaches from the promoter, allowing the promoter space to allow RNA to polymerize. The enzyme can combine with the promoter to start the transcription of the fatty acid synthesis gene and then regulate the rate of fatty acid synthesis according to the concentration of intracellular Malonyl-CoA. FapR has the property of binding to FapO through gel retardation experiments, and it has been confirmed that the concentration of Malonyl-CoA will affect the proportion of FapR and FapO binding. As the concentration of Malonyl-CoA increases, the FapR that can bind to FapO decreases significantly.

Zhang’s research group used the natural FapR and FapO of *Bacillus subtilis* to construct the Malonyl-CoA sensor (Zhang et al., 2012) and used the sensor to regulate the artificially constructed fatty acid synthesis pathway to increase fatty acid production by 33–34% of the yield (Liu et al., 2015b). After that, David (David et al., 2016) adjusted the position and copy number of FapO in the promoter −35/−10 region or −10 downstream to reduce the energy loss during the synthesis of 3-HP by *S. cerevisiae* and increase the yield of 3-HP by 10 times. Yang et al. (2015) used antisense RNA technology to suppress specific genes’ expression in the fatty acid synthesis pathway. The intracellular Malonyl-CoA concentration increased by 4.5 times, and the downstream products of Malonyl-CoA, 4-hydroxycoumarin, resveratrol, and naringenin. The output increased by 2.53, 1.70, and 1.53 times, respectively. Because of the previous research ideas, the low concentration of Malonyl-CoA restricts poly(3HP) synthesis, but the concentration is too high to poison and kills the bacteria. Intention to use the Malonyl-CoA sensor to simultaneously regulate intracellular Malonyl-CoA concentration, solving cell density and poly(3HP) production.

## Life Cycle Assessment for the Production of Poly(3HP) From Corn Oil

**FIGURE 4:**
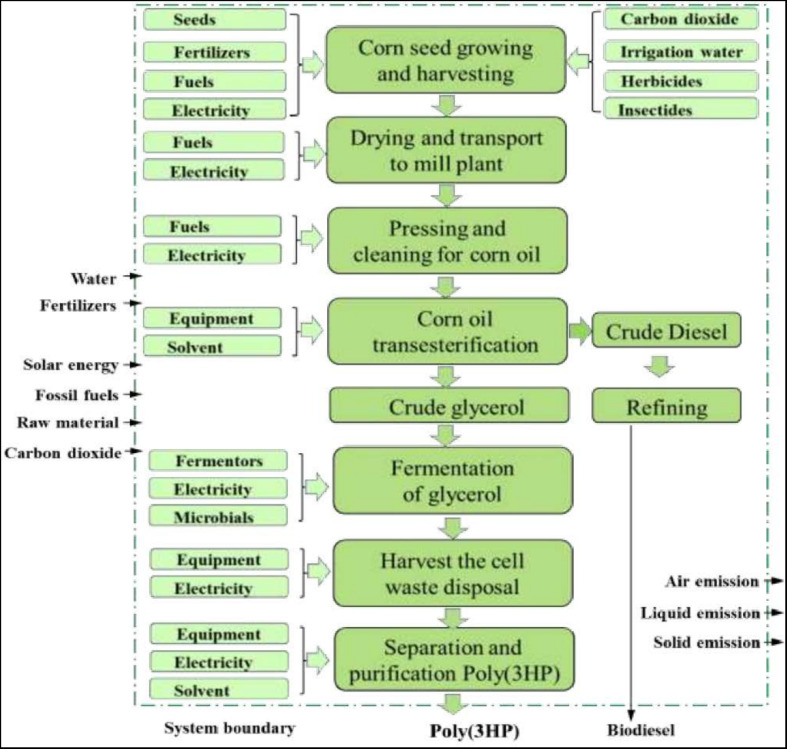
Poly(3HP) production with biodiesel manufacturing side product-glycerol.

Life Cycle Assessment (LCA) is a method designed to assess a product’s impacts or processes on the environment (Rebitzer et al., 2004). LCA is an architecture that can evaluate a product’s environmental impacts throughout its life, starting from the extraction of raw materials from growth and ending at the waste products being disposed to the earth. Many researchers have carried out LCA investigations on PHAs polymer’s biosynthesis (Khoo et al., 2010; Heimersson et al., 2014). The LCA of bioplastics has revealed that PHAs polymer’s production and application are more environmentally friendly than synthetic polymer in consideration of energy consumption and greenhouse gas emissions (Ali and Jamil, 2016). The abundant availability of corn in the world is used to produce corn oil, and the LCA to produce poly(3HP) from corn oil has been shown in Figure 4.

The corn growing need corn seed, carbon dioxide, fertilizer, water, electricity, and fuel (diesel, propane, and gasoline) used by the farmer. The atmospheric carbon dioxide takes up through the photosynthesis process, irrigation water, and pesticides. Then harvest and transport of corn to the corn mill plant, transesterification of corn oil into crude glycerol, fermentation and purification of glycerol, and downstream of biopolymer poly (3HP) to poly(3HP) (Trirahayu, 2020). On the output side, emissions such as dinitrogen oxide, nitrates, and phosphates were considered. Glycerol is widely used as a raw material to manufacture various cosmetics, pharmaceuticals, and packaging. The production of glycerol can use corn oil through several processes such as transesterification, saponification with NaOH, and fat splitting (Marx, 2016). The production of biodiesel with glycerol as a waste product is term as transesterification, and the raw material used in this process is corn oil.

## Physical/Material Properties of Poly(3HP)

Poly(3HP) homopolymer is a well-known biodegradable, thermoplastic polyester with high tensile strength, elongation at breaking, and high moisture permeability. Poly(3HP) possess a melting peak temperature of 79.1^*o*^C in its pure state and about 77.7^*o*^C in blends with the Poly(3HP) content ranging from 50–90%, a glass transition temperature of −20, and a fusion enthalpy value of 64J/g, as shown in Table 1 (Andreeßen et al., 2014b). Although poly(3HP) has received focus to be environmentally degradable material, it has some limitations due to brittleness (high crystallinity), limited processability, and high hydrophobicity (Catiker and Basan, 2017). Some studies have been conducted on poly(3HP) blends to eliminate these abnormalities (He et al., 2000). Similarly, various poly(3HP) copolymers have been examined by blending their favorable material properties with other components (Andreeßen and Steinbüchel, 2010; Zhou et al., 2011).

Some polymer blends with poly (3-HP) are becoming more critical and efficient routes of developing new polymeric material in the polymer industry. Various polymer blends have been researched and further applied in some fields, such as scaffold material in tissue engineering and polymeric matrix for drug delivery.

Polymer blends and polymer thermal degradation of poly (3-HP) have been investigated by applying one or more of the following techniques such as thermogravimetry (TGA), differential thermal analysis (DTA), dynamic mechanical, thermal analysis (DMTA), differential scanning calorimetry (DSC), and nuclear magnetic resonance (NMR) (Catiker and Basan, 2017). The miscibility and thermal degradation of [poly(3HP)] and poly-β-alanine (poly (BA) have been studied thoroughly. Catiker and Basan (2017) studied the miscibility of poly(3HP) and poly(BA) and found out that blends of these two components are miscible in the amorphous phase. The miscibility occurred because of the suppression of the crystallinity of both elements in the blends. The thermal degradation of poly(3HP) and poly(BA) was examined, and the results have shown that combinations of these components turned to decompose thermally via fast and single-step degradation (Catiker and Basan, 2017).

Furthermore, the miscibility of biodegradable blends of high molecular weight poly (ethylene oxide) [poly (EO)] with poly(3HP) and poly (3-hydroxybutyric acid) have also been studied by He et al. (2000). However, binary blends of low molecular weight of poly(EO) (*M*_V_ = 2.0 × 10^4^gmol^–1^) with poly(3HP) has been reported to be miscible according to some experiments using DSC measurements.

## Importance Materials of Poly(3HP)

Overall, various copolymers and block polymers have a more extensive range of industry and medicine applications due to their barrier properties and mechanical strength than other bioplastics such as polylactic acid. Despite its intrinsic brittleness, much progress has been made through the formulation of poly(3HP) with tailored additives and blends, leading to improved mechanical strength and suitable processability via extrusion or injection molding (Torres-Martínez et al., 2019).

### Medical Applications of Poly (3-HP)

#### Scaffold Material in Tissue Engineering

Recently, biodegradable and biocompatible polymeric scaffolds have been applied in tissue regeneration functions. Cortizo et al. (2008) experimented with developing a polymeric-based scaffold for bone regeneration using two polyesters (poly-β-propiolactone and poly-ε-caprolactone) and two polyfumarates (polydiisopropyl fumarate and polydicyclohexyl fumarate). Their studies showed that poly-β-propiolactone (PBPL), currently known as poly(3HP), was found out to support osteoblastic growth through adhesion. Growth and differentiation of two osteoblastic cell lines (mouse calvaries-derived MC3T3E1 cells and UMR106 rat osteosarcoma cells) after biodegradation and cytotoxicity. It was known that poly (3-HP) offers the best rougher and more porous surface. Under acellular conditions, poly (3-HP) was degraded by a hydrolytic mechanism. Well-defined actin fibers of osteoblasts were developed without any evidence of cytotoxicity when growing on films. It was observed that the number of UMR106 osteoblasts that adhered to poly (3-HP)-the based film was higher compared to cells attached to the two polyfumarates matrices. Furthermore, there was a significant increase in the proliferation of UMR106 cells on polyester-derived scaffolds (poly(3HP) and Poly-ε-caprolactone) (Cortizo et al., 2008). Fix a general diagram for tissue engineering

#### Polymeric Matrix for Drug Delivery

Poly(3HP) film can be applied in drug delivery system requiring a sustained and controlled delivery mechanism. An experiment conducted by Cortizo et al. (2001) proved the release kinetics of a complex of vanadium (IV) with aspirin (VOAspi) on films from polymers of different molecular weights and as well as with variable drug load. After 7 days, a sustained release of vanadium on films of other polymers, two contributing factors, (a) diffusion of the drug, and (b) degradation of the poly(3HP) film, was postulated. Analyzing the experimental data of the drug’s diffusion coefficient (VOAspi) using a diffusion model, VOAspi does not show strong interaction with poly (3-HP), and UMR106 osteosarcoma cells show no anticarcinogenic effects of VOAspi releases from the film.

Moreover, cell proliferation was inhibited by the VOApi-poly(3HP) in a dose-response manner and induced half of the thiobarbituric acid reactive substances formation (TBARS), an index of lipid peroxidation, as compared to that with free VOAspi in solution. There was no cytotoxicity generated by the poly(3HP) film as cell growth, and TBARS was evaluated. The poly(3HP) film- embedded with VOASpi retained an antiproliferative effect evidencing lower cytotoxicity than the free drug (Cortizo et al., 2001).

## Conclusion and Future Perspectives

As mentioned in this review, considering the case of recent advances in poly(3HP) biosynthesis by researchers who have established an active poly(3HP) biosynthetic pathway through genetic engineering techniques; thus the Malonyl-CoA and glycerol pathways have been modified to utilize an abundant and inexpensive carbon source to improve the synthesis of poly(3HP) to some appreciable production. In terms of the efficiency of poly(3HP) synthesis, the new β-alanine pathway is optimized to reduce the production costs, and it is now the hotspot in poly(3HP) biosynthesis. Furthermore, in the case of poly(3HP) production in microbial host chassis, there are many factors to the generative design of an appropriate production strain, ranging from the selection of the microbial host, the pathway, and the enzymes to the genetic engineering of the cell for improved process through the biosynthetic pathway of production and the ability to accommodate/tolerate metabolic stress facilitated by intermediate, and the final products (Figure 1). Most of the studies described in the review have targeted the highest titers recorded so far by these cell factories, of which a recombinant strain of *E. coli* under optimal conditions accumulated the highest CDW of 21.8 g/L containing 46.4% of poly(3HP) without the addition of any expensive precursor in an aerobic fed-batch fermentation (Wang et al., 2013).

The impressive body of the research on biosynthetic pathways construction has identified numerous challenges such as (a) addition of important coenzymes and poly(3HP) structures similar to precursors to increase production costs, (b) the loss of plasmids in genetically engineered strains result in lower titers in the final product of poly(3HP), and (c) in the poly(3HP) biosynthetic pathway, the of related intermediates is too high, which causes cytotoxicity. These challenges are yet to be resolved, and with the development of gene editing and expression modification technologies, has been gradually applied in the biochemical industry; as reported by Gao et al. (2014), the use of genomic integration technology has effectively increased the titer of poly(3HP) in the glycerol and Malonyl-CoA pathways, respectively (Gao et al., 2014). Also, the negative feedback regulation mechanism of biosensor developed by Zhang et al. (2012), the key to MCR enzyme activity control factor, and design a negative feedback regulatory pathway to regulate the enzyme activity and cell proliferation ability of MCR at a higher level (Zhang et al., 2012). Again, the use of gene knockout to reduce the production of metabolites unrelated to the final product.

Finally, more powerful and advanced engineering techniques/tools are continually being developed; notably, the recent developments of 3-HP and Malonyl-CoA biosensors can be used in combination with adaptive laboratory evolution for identifying as-of-yet unknown mechanism for further fine-tuning of production strains.

In conclusion, the advanced development of synthetic biology and metabolic engineering should be geared toward designing large combinatorial cell factory libraries using only renewable and inexpensive carbon sources, thereby promoting the industrialization of economic large scale production of poly(3HP).

## Author Contributions

AGA and HC performed the literature search and wrote the manuscript. HY and CWL revised and edited the English literature. GS and CLL critically revised and provided conceptual comments. All authors reviewed the manuscript.

## Conflict of Interest

The authors declare that the research was conducted in the absence of any commercial or financial relationships that could be construed as a potential conflict of interest.
